# Supplementary material for: Gestational Diabetes Mellitus Subtypes Derived by Clustering Analysis Show Heterogeneity in Glucometabolic Parameters Already at Early Pregnancy
Source: Nutrients. 2025 Oct 16;17(20):3252. doi: 10.3390/nu17203252 (PMC12566870; doi:10.3390/nu17203252)
Supplement: Supplementary file 1 [file nutrients-17-03252-s001.zip › nutrients-3876724-supplementary.pdf]

Table S1: Dietary habits at early pregnancy of the study population

	NGT (n=745)	CL1 (n=10)	CL2 (n=58)	CL3 (n=86)
Milk (ml/d)	100 (18-200)	100 (100-200)	100 (21-200)	100 (43-200)
Water (l/d)	1 (1-5)	5 (1-5)	1 (1-4)	1 (1-4)
Non-alcoholic beverages (ml/d)	173 (52-403)	289 (54-398)	134 (63-410)	104 (50-350)
Coffee (ml/d)	13 (0-150)	16 (0-150)	13 (0-150)	6 (0-118)
Tea (ml/d)	113 (13-300)	150 (23-300)	150 (5-450)	137 (13-450)
Bread (g/d)	84 (50-150)	107 (37-147)	105 (54-153)	96 (50-146)
Rice, couscous, bulgur (g/d)	16 (13-32)	23 (8-32)	32 (7-75)	24 (13-32)
Noodles (g/d)	27 (11-27)	6 (4-13)	27 (6-27)	13 (6-27)
Potatoes (g/d)	46 (25-83)	30 (15-41)	59 (38-107)	38 (18-56)
Pizza (g/d)	13 (6-31)	10 (6-27)	16 (6-27)	13 (4-31)
Breakfast cereals (g/d)	2 (0-9)	2 (0-5)	2 (0-6)	1 (0-6)
Legumes (g/d)	7 (3-16)	7 (1-23)	13 (5-16)	13 (3-16)
Vegetables (g/d)	88 (32-182)	42 (31-72)	107 (18-150)	64 (33-169)
Fruits (g/d)	238 (123-353)	151 (121-300)	274 (150-459)	234 (143-360)
Butter and magarine (g/d)	2.5 0.5-10.0)	2.0 (0.1-4.3)	2.5 (0.0-10)	2.0 (0.5-9.5)
Cheese (g/d)	12.9 (3.2-30.0)	4.8 (1.7-26.3)	12.9 (4.3-30)	15 (2.9-30)
Cream Cheese (g/d)	3.2 (0.3-15)	2.4 (0.4-21.4)	3.2 (0.0 (13.4)	3.2 (0.3-15.0)
Curd cheese, soured milk, yoghurt (g/d)	43 (9-100)	13 (7-38)*	39 (0-100)	43 (18-100)
Eggs (g/d)	13 (5-30)	26 (13-60)	26 (9-50)	13 (5-30)
Meat (g/d)	13 (5-26)	11 (4-26)	26 (9-26)	13 (5-26)
Meat products (g/d)	9 (1-20)	9 (1-17)	12 (2-31)	7 (0-17)
Poultry (g/d)	16 (7-32)	32 (18-32)	13 (3-32)	16 (5-32)
Fish (g/d)	8 (1-18)	8 (3-19)	11 (3-19)	4 (1-16)
Fast Food (g/d)	10 (0-25)	20 (11-29)	20 (5-35)	10 (0-25)
Sweat spreads (g/d)	2.9 (1-9)	4 (1-7)	3 (1-7)	2 (1-10)
Sweets (g/d)	35 (19-67)	30 (12-199)	31 (13-79)	27 (15-59)
Salty snacks (g/d)	4 (2-10)	4 (2-13)	4 (2-7)	3 (1-7)

Data are mean median (IQR) for women remaining normal glucose tolerant (NGT) vs. patients developing gestational diabetes (GDM) categorized into clusters (CL 1 – 3). All groups were compared with NGT, and Dunnett post hoc tests were used to adjust for multiple testing.

* $p<0.05$ vs. NGT
